# Efficacy of benralizumab for patients with severe eosinophilic asthma: a retrospective, real-life study

**DOI:** 10.1186/s12890-020-01248-x

**Published:** 2020-08-03

**Authors:** Takanori Numata, Hanae Miyagawa, Saiko Nishioka, Keitaro Okuda, Hirofumi Utsumi, Mitsuo Hashimoto, Shunsuke Minagawa, Takeo Ishikawa, Hiromichi Hara, Jun Araya, Kazuyoshi Kuwano

**Affiliations:** Division of Respiratory Diseases, Department of Internal Medicine, The Jikei University School of Medicine, 3-25-8, Nishi-Shimbashi, Minato-ku, Tokyo, 105-8461 Japan

**Keywords:** Eosinophilic asthma, Benralizumab, Eosinophilic chronic rhinosinusitis, Global evaluation of treatment effectiveness

## Abstract

**Background:**

Benralizumab, an anti-interleukin-5 (IL-5) receptor α monoclonal antibody, significantly reduces the number of annual exacerbations and oral corticosteroid (OCS) maintenance doses for patients with severe eosinophilic asthma (SEA). However, few studies on the efficacy of this biologic in real life are available. The aim was to elucidate the efficacy of benralizumab by evaluating changes in clinical parameters after benralizumab treatment in patients with SEA.

**Methods:**

From July 2018 to December 2019, 24 Japanese patients with SEA received benralizumab at Jikei University Hospital. We retrospectively evaluated the patients’ characteristics, parameters, numbers of exacerbations and maintenance OCS doses.

**Results:**

Among the 24 patients, eleven patients had received mepolizumab treatment and were directly switched to benralizumab. The peripheral blood eosinophil and basophil counts significantly decreased after benralizumab treatment regardless of previous mepolizumab treatment. Pulmonary function, Asthma Control Test scores, the numbers of annual exacerbations and maintenance OCS doses in patients without previous mepolizumab treatment tended to improve without significant differences. Fourteen patients (58%) were responders according to the Global Evaluation of Treatment Effectiveness (GETE) score. The proportion of GETE responders among patients with aspirin-exacerbated respiratory disease (AERD) tended to be lower than that among patients without AERD (*p* = 0.085). After benralizumab treatment, the change in the forced expiratory volume in 1 s from baseline was 200 ml or greater in eight patients (33%), including three patients who were switched from mepolizumab.

**Conclusion:**

Benralizumab treatment improved and controlled asthma symptoms based on the GETE score.

## Background

Bronchial asthma is a common and chronic respiratory disease affecting 300 million people worldwide [1]. The prevalence of severe or difficult-to-treat asthma is approximately 3–10% [2, 3]. Severe and uncontrolled asthma has been reported to be associated with diminished health-related quality of life and high healthcare costs [4, 5].

The anti-interleukin-5 receptor α (IL-5RA) antibody benralizumab exerts antibody-dependent cell-mediated cytotoxicity (ADCC), the process by which natural killer cells cause eosinophil apoptosis, with rapid and nearly complete depletion in the peripheral blood. Benralizumab has been demonstrated to be an effective therapy for patients with severe eosinophilic asthma (SEA) as it reduces annual exacerbation rates and maintenance oral corticosteroid (OCS) doses and improves pulmonary function [6–9]. Although mepolizumab, an anti-IL-5 monoclonal antibody, has shown clinical efficacy for patients with SEA in Japan [10, 11], little evidence is available regarding benralizumab therapy for patients with severe asthma [12].

We therefore conducted this single-center retrospective study to elucidate the efficacy of benralizumab in a real-life setting.

## Methods

### Subjects

From July 2018 to December 2019, 24 Japanese patients with SEA received benralizumab injections (30 mg every 4 weeks for the first three injections and every 8 weeks for the subsequent injections) at Jikei University Hospital, Tokyo, Japan. All asthma patients were diagnosed by respiratory physicians based on Japanese guidelines [13] or the Global Initiative of Asthma (GINA) guidelines [4]. Severe asthma was defined as requiring a high dose of inhaled corticosteroids (ICSs) plus at least one of the following additional control measures: long-acting β-2 agonists (LABAs), long-acting muscarinic antagonists (LAMAs), leukotriene receptor antagonists (LTRAs), a xanthine derivative and a daily OCS [3, 4, 13]. Xanthine derivatives are routinely prescribed in Japan because the Japanese guideline for the treatment of adult asthma recommends the use of xanthine derivatives in the setting of insufficient control with conventional control measures. SEA was defined as severe asthma with eosinophilic airway inflammation, which was defined as a peripheral blood eosinophil count ≥300/μl.

The present study was approved by the Ethics Committee of Jikei University [30–319 (9340)]. Based on the ethical guidelines of Jikei University, informed consent was not necessary for this retrospective study, and we performed opt-out consent on the website of our hospital. The benralizumab prescription rule was based on the Pharmaceuticals and Medical Devices Agency in Japan. The inclusion criteria for initiating benralizumab treatment in patients were as follows: 1. the patient had at least two exacerbations requiring OCS treatment in the previous year or before the introduction of mepolizumab; 2. if the patient did not receive mepolizumab treatment, the patient’s blood eosinophil count was at least 150/μl at baseline or ≥ 300/μl in the previous year; or 3. the patient received OCS maintenance therapy or another biologic (omalizumab or mepolizumab) regardless of the number of exacerbations or the blood eosinophil count.

### Data collection and evaluation

We retrospectively examined the following characteristics: sex, age, comorbidities of eosinophilic diseases, smoking status, body mass index (BMI), baseline treatments including biologics, and the duration of asthma. We examined and evaluated the following parameters at baseline and at the final follow-up after 4 months: peripheral blood eosinophil and basophil counts, serum IgE, fractional exhaled nitric oxide (FeNO), the Asthma Control Test (ACT) score, pulmonary function test results [the forced vital capacity (FVC), forced expiratory volume in 1 s (FEV_1_), FEV_1_/FVC, %FEV_1_ and % peak expiratory flow (%PEF)], and daily OCS maintenance doses as prednisone equivalents (mg). A change of 200 ml or greater was adopted as a significant change in the FEV_1_ [12, 14–16]. The FeNO level was measured using a NIOX VERO™ device (Aerocrine AB, Stockholm, Sweden) with a 50 ml/s flow rate according to the American Thoracic Society/European Respiratory Society recommendations [17]. The number of annual exacerbations of asthma symptoms requiring systemic CS was defined as the total number of exacerbations × 12/the total duration of the observation period (months). Furthermore, we evaluated changes from baseline in these parameters. To evaluate clinical efficacy, we utilized the ACT score and the Global Evaluation of Treatment Effectiveness (GETE) score; the ACT score is clinically useful as a simple scoring system, and scores of 20–25 are classified as well-controlled asthma [4]. The minimal clinically important difference (MCID) was an ACT score of three points [18]. The GETE score was evaluated based on symptom severity, medication use and pulmonary function tests at the final follow-up after at least 4 months of benralizumab treatment [19]. The GETE score has five classifications: excellent, good, moderate, poor and worsening. A responder is defined as a patient with a good/excellent response when treated with benralizumab. The GETE score after mepolizumab treatment was used if the overall evaluation did not change for the patients switched from mepolizumab.

The primary endpoint was the GETE score. We performed a subgroup analysis based on previous mepolizumab treatment with eosinophilic chronic rhinosinusitis (ECRS) or chronic rhinosinusitis with nasal polyps (CRSwNP) and with aspirin-exacerbated respiratory disease (AERD) because these factors affect the evaluation of clinical efficacy. The secondary endpoints included each parameter, each biomarker, changes in the number of asthma exacerbations and in daily CS doses, and the proportion of patients with a change in FEV_1_ from baseline (≥ 200 ml). To examine sinusitis symptoms and findings, we utilized nasal discharge, nasal congestion and olfactory loss reported in the medical records.

### Statistical analyses

All statistical analyses were performed using StatView version 5 (SAS Institute, Inc., Cary, NC, USA), and a post-hoc power analysis was performed with EZR (version 1.37, Saitama Medical Center, Jichi Medical University, Saitama, Japan) [20], which is a graphical user interface for R (version 3.4.1, The R Foundation for Statistical Computing, Vienna, Austria). All values are expressed as the mean ± standard deviation (SD). A *p*-value < 0.05 was considered statistically significant. To determine the characteristics of the responders, we statistically analyzed univariate models of patient characteristics, such as sex, age, BMI, peripheral blood eosinophil and basophil counts, previous mepolizumab treatment, and comorbidities. The factors associated with patient characteristics were analyzed using the Mann-Whitney *U* test, Fisher’s exact test, or the Wilcoxon signed-rank test (univariate model). Because the number of patients in the present study was small, we re-evaluated the clinical parameters, the percentage change in the number of annual exacerbations and maintenance CS doses with a post hoc power analysis (α-error < 0.05, cut-off 0.80). Furthermore, logistic regression analysis was performed to evaluate the identified characteristics of the responders (multivariate model), including the peripheral blood eosinophil count (≥ 300 /μl), ECRS or CRSwNP [11] as a comorbidity and other variables that achieved *p* < 0.20 in the univariate model.

## Results

### Patient characteristics

Nine men and 15 women received benralizumab treatment with eight doses (median). The characteristics of the 24 patients are shown in Table 1. Among them, 11 had directly switched to benralizumab from mepolizumab treatment based on asthma symptoms (*n* = 2), the interval between hospital visits (*n* = 6) and discussions with physicians (*n* = 3) (Additional file 1). No significant differences in patient characteristics were found between the patients with and without previous mepolizumab treatment except for eosinophilic otitis media, which was frequently present in patients with previous mepolizumab treatment. In addition, we assessed the patients’ characteristics in the following subgroups (Additional file 2): males, females, AERD (−) and AERD (+). We showed that the %FEV_1_, %PEF and ACT score were significantly lower in males than in females using post-hoc power analysis. In addition, the AERD (+) group showed significantly lower ACT scores and tended to have a lower %FEV_1_ at baseline than the AERD (−) group (Additional file 2).

**Table 1:** Patient characteristics at baseline (*n* = 24)

	all patients (*n* = 24)	previous mepolizumab treatment (−) (*n* = 13)	previous mepolizumab treatment (+) (*n* = 11)	*p* value between two groups
male, n (%)	9 (38)	4 (31)	5 (45)	0.68^†^
age (years), mean (SD) (range)	57.5 (13.4) (20–75)	56.7 (15.8) (20–75)	58.4 (10.6) (38–72)	0.91^‡^
disease duration, (years), mean (SD) (range)	21.3 (12.5) (4–54)	21.2 (14.2) (4–54)	21.5 (10.7) (8–36)	0.68^‡^
body mass index (kg/m^2^), mean (SD)	23.4 (4.8)	24.6 (5.1)	21.9 (4.1)	0.19^‡^
smoking (never/former), n	18 / 6	9 / 4	9 / 2	0.65^†^
initial treatments use				
—ICS/LABA, n (%)	24 (100)	13 (100)	11 (100)	–
—ICS dose (μg), mean (SD), budesonide equivalent	1381 (448)	1411 (518)	1345 (370)	0.66^‡^
—LAMA, n (%)	14 (58)	7 (54)	7 (64)	0.70^†^
—LTRA, n (%)	20 (83)	11 (85)	9 (82)	> 0.99^†^
—xanthine derivative, n (%)	17 (71)	9 (69)	8 (73)	> 0.99^†^
—maintenance therapy of OCS, n (%)	8 (33)	6 (46)	2 (18)	0.21^†^
—daily dose of OCS^a^ (mg), mean (range)	5.6 (1.0–15)	6.1 (1.0–15)	4.3 (2.5–6)	0.74^‡^
comorbidities				
—ECRS, n (%)	20 (83)	10 (77)	10 (91)	0.60^†^
—EOM, n (%)	11 (46)	3 (23)	8 (73)	0.038^†^
—AERD, n (%)	7 (29)	6 (46)	1 (10)	0.08^†^
—EGPA, n (%)	4 (17)	1 (8)	3 (27)^b^	0.30^†^
—atopic dermatitis, n (%)	1 (4)	1 (8)	0 (0)	> 0.99^†^
previous biologics				
—omalizumab, n (%) / median (range) (month)	5 (21) / 11 (3–88)	1 (8) / 3 (3)	4 (40) / 15.5 (4–88)	0.14^†^ / 0.16^‡^
—mepolizumab, n (%) / median (range) (month)	11 (46) / 21 (5–35)	–	11 (100) / 21 (5–35)	–
—dupilumab, n (%) / median (range) (month)	1 (4) / 6 (6)	1 (8) / 6 (6)	–	–
number of benralizumab injections, median (range)	8 (2–11)	8 (2–10)	7 (4–11)	0.98^‡^
observation period (months), median (range)	11.5 (4–17)	11 (4–16)	14 (4–17)	0.79^‡^

Data are presented as n (%) or mean (standard deviation), unless otherwise stated

*Abbreviations*: *SD* standard deviation, *ICS* inhaled corticosteroid, *LABA* long-acting β-2 agonist, *LAMA* long-acting muscarinic antagonist, *LTRA* leukotriene receptor antagonist, *OCS* oral corticosteroids, *ECRS* eosinophilic chronic rhinosinusitis, *EOM* eosinophilic otitis media, *AERD* aspirin-exacerbated respiratory disease, *EGPA* eosinophilic granulomatosis with polyangiitis

^†^Fisher’s exact test, ^‡^Mann-Whitney *U* test,

^a^prednisone equivalents dose

^b^All three patients with EGPA received 100 mg of mepolizumab injection

### Clinical efficacy

The changes in clinical parameters and biomarkers are shown in Table 2. The peripheral blood eosinophil and basophil counts significantly decreased. However, no significant differences in the changes in pulmonary function, FeNO, the ACT score, the number of annual exacerbations or maintenance CS doses from baseline were found between all patients or in the presence or absence of previous mepolizumab treatment. The %FVC, %FEV_1_ and FEV_1_ were reevaluated using post-hoc power analysis, and no significant difference was found before and after benralizumab treatment regardless of previous mepolizumab treatment. The ACT score tended to increase in all patients (*n* = 24) and in the group without previous mepolizumab (*n* = 13) treatment without significant differences (Table 2). Furthermore, the total number of patients with a final ACT score = 25 (well-controlled asthma) or an ACT score increase ≥3 (significant change) was 11 (85%) among the patients without previous mepolizumab treatment (*n* = 13) (data not shown). We determined the %FEV_1_ and ACT score before and after benralizumab treatment in the AERD (−) and (+) groups (Additional file 3). Although the %FEV_1_ tended to improve in the AERD (−) group, no significant difference in the %FEV_1_ was observed before and after benralizumab treatment in the AERD (+) group.

**Table 2:** Change from baseline to last follow-up in asthma patients with or without previous mepolizumab treatment

	all patients(*n* = 24)			previous mepolizumab treatment (−) (*n* = 13)			previous mepolizumab treatment (+) (*n* = 11)			*P* value between two groups at baseline
	baseline	last follow up	*P* value	baseline	last follow up	*P* value	baseline	last follow up	*P* value	*P* value
peripheral blood eosinophil counts (/μl)	292 (312)	0 (0)	< 0.0001^†^	458 (338)	0 (0)	0.0015^†^	95 (88)	0 (0)	0.0033^†^	0.0005^†^
peripheral blood basophil counts (/μl)	35 (27)	7 (9)	< 0.0001^†^	35 (26)	6 (5)	0.0015^†^	35 (30)	8 (13)	0.0033^†^	0.95
serum IgE (IU/ml)	232(198)	325 (674)	0.70	263 (186)	232 (225)	0.48	196 (213)	436 (980)	0.88	0.27
FeNO (ppb)	59 (39)	67 (53)	0.41	64 (43)	71 (58)	0.70	52 (32)	61 (47)	0.40	0.53
%FVC (%)	94.9 (14.9)	97.9 (14.3)	0.11	96.1 (16.6)	101.5 (14.2)	0.028	93.4 (13.2)	93.7 (13.9)	0.76	0.52
%FEV_1_ (%)	78.4 (21.9)	81.9 (21.2)	0.017	79.8 (26.2)	84.6 (24.1)	0.006	76.8 (16.6)	78.8 (17.8)	0.53	0.98
FEV_1_/FVC (%)	67.2 (10.8)	68.4 (12.3)	0.29	67.1 (12.1)	67.9 (12.5)	0.35	67.2 (9.7)	69.0 (12.7)	0.53	0.75
FEV_1_ (ml)	1978 (596)	2057 (606)	0.07	1936 (783)	2053 (778)	0.017	2026 (275)	2061 (346)	0.96	0.04
%PEF (%)	83.9 (24.8)	84.1 (24.4)	0.72	86.6 (29.9)	88.0 (27.5)	0.70	80.6 (17.9)	79.0 (19.8)	0.22	0.88
ACT (pts)	19.2 (4.8)	20.3 (4.9)	0.08	17.8 (5.9)	20.2 (5.5)	0.08	20.9 (1.8)	20.3 (4.3)	0.51	0.51
number of annual exacerbations	2.8 (3.4)	1.8 (3.2)	0.28	3.6 (4.0)	1.7 (3.5)	0.10	1.8 (2.3)	2.0 (2.8)	0.67	0.31
prednisolone equivalent dose (mg/day)	5.6 (4.2)	3.3 (2.5)	0.11	6.1 (4.7)	3.0 (2.6)	0.11	4.3 (2.5)	4.3 (2.5)	–	0.74

Data are presented as the mean (standard deviation) and were analyzed using the Wilcoxon signed rank test

^†^*P* values with sufficient power that were re-evaluated by a post-hoc power analysis

*Abbreviations*: *FeNO* fractional exhaled nitric oxide, *FVC* forced vital capacity, *FEV*_*1*_ forced expiratory volume in one second, *PEF* peak expiratory flow, *ACT* Asthma Control Test, *ppb* parts-per-billion

**Fig. 1:**
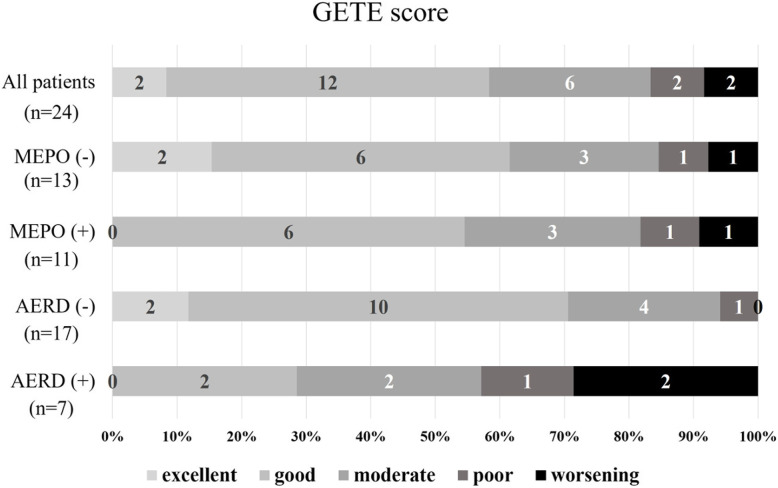
GETE scores. The GETE scores for the following five settings: all patients (*n* = 24, 1st (upper)), without previous mepolizumab treatment (*n* = 13, 2nd), with previous mepolizumab treatment (*n* = 11, 3rd), without AERD (*n* = 13, 4th) and with AERD (*n* = 11, 5th (bottom)). Except for the AERD group, the response rates to benralizumab were approximately 60–70%. Abbreviations: GETE, Global Evaluation of Treatment Effectiveness, AERD, aspirin-exacerbated respiratory disease

We show the GETE scores of all patients, the previous mepolizumab treatment (−) / (+) groups and the AERD (−) / (+) groups in Fig. 1. The total responder rate to benralizumab treatment was 58% (14 patients), including good and excellent responses. Regardless of previous mepolizumab treatment, the response rate was approximately 60%. The response rate in the AERD (−) group was higher than that in the AERD (+) group [71% (12/17) vs 29% (2/7), *p* = 0.085].

We compared baseline clinical parameters and comorbidities between the responders and nonresponders to benralizumab treatment (Table 3). A significant difference in the %FVC at baseline was found between these two groups. No significant differences in clinical parameters at baseline were observed between the responders and nonresponders in the previous mepolizumab treatment (−) and (+) groups. To further examine the involvement of baseline clinical parameters in the efficacy of benralizumab, we analyzed changes in the FEV_1_ of ≥200 ml and < 200 ml (Table 3). The change in the FEV_1_ from baseline was 200 ml or greater in eight patients (33%), including three patients who were switched from mepolizumab treatment (Table 3). The %FVC and %FEV_1_ at baseline were significantly lower in patients with a change in the FEV_1_ ≥ 200 ml than in those with a change in the FEV_1_ < 200 ml.

**Table 3:** Patient characteristics based on the clinical efficacies

at baseline	GETE ≥ good (*n* = 14)	GETE ≤ moderate (*n* = 10)	*p* value	ΔFEV_1_ ≥ 200 ml (*n* = 8)	ΔFEV_1_ < 200 ml (*n* = 16)	*p* value
sex (M/F), n	4 / 10	5 / 5	0.40	5 / 3	4 / 12	0.099
age (years)	57.6 (12.1)	57.2 (15.7)	0.79	51.6 (15.8)	60.4 (11.5)	0.15
—MEPO (−) (*n* = 13)	56.9 (12.6) (*n* = 8)	56.4 (21.8) (*n* = 5)	0.71	52.8 (19.4) (*n* = 5)	59.1 (14.0) (*n* = 8)	0.51
—MEPO (+) (*n* = 11)	58.7 (12.6) (*n* = 6)	58.0 (9.0) (*n* = 5)	0.85	49.7 (10.2) (*n* = 3)	61.6 (9.2) (*n* = 8)	0.098
BMI (kg/m^2^)	22.0 (4.2)	25.3 (5.1)	0.09	24.7 (5.5)	22.7 (4.4)	0.46
—MEPO (−) (*n* = 13)	22.6 (5.0) (*n* = 8)	27.7 (3.8) (*n* = 5)	0.079	26.2 (5.0) (*n* = 5)	23.5 (5.3) (*n* = 8)	0.38
—MEPO (+) (*n* = 11)	21.2 (2.9) (*n* = 6)	22.8 (5.4) (*n* = 5)	0.58	22.1 (6.3) (*n* = 3)	21.8 (3.5) (*n* = 8)	0.84
bEOS (/μl)	306 (216)	271 (426)	0.20	407 (424)	234 (234)	0.27
—MEPO (−) (*n* = 13)	437 (183) (*n* = 8)	492 (531) (*n* = 5)	0.66	565 (472) (*n* = 5)	391 (236) (*n* = 8)	0.66
—MEPO (+) (*n* = 11)	132 (104) (*n* = 6)	50 (35) (*n* = 5)	0.20	143 (127) (*n* = 3)	77 (71) (*n* = 8)	0.31
bBASO (/μl)	33 (29)	38 (27)	0.46	49 (40)	28 (16)	0.34
—MEPO (−) (*n* = 13)	28 (18) (*n* = 9)	46 (35) (*n* = 5)	0.38	41 (37) (*n* = 5)	31 (19) (*n* = 8)	0.77
—MEPO (+) (*n* = 11)	40 (40) (*n* = 6)	29 (13) (*n* = 5)	0.86	61 (51) (*n* = 3)	26 (13) (*n* = 8)	0.31
FeNO (ppb)	70 (42)	41 (26)	0.08	70 (39)	53 (38)	0.26
—MEPO (−) (*n* = 13)	74 (49) (*n* = 8)	47 (30) (*n* = 5)	0.27	66 (42) (*n* = 5)	63 (48) (*n* = 8)	0.71
—MEPO (+) (*n* = 10)^a^	64 (33) (*n* = 6)	33 (22) (*n* = 4)^a^	0.11	75 (43) (*n* = 3)	42 (23) (*n* = 7)^a^	0.25
%FVC (%)	99.1 (17.6)	88.9 (7.2)	0.04^†^	83.8 (9.9)	100.4 (14.0)	0.005^†^
—MEPO (−) (*n* = 13)	101.0 (19.6) (*n* = 8)	88.3 (5.9) (*n* = 5)	0.04^†^	84.6 (12.8) (*n* = 5)	103.3 (15.1) (*n* = 8)	0.04
—MEPO (+) (*n* = 11)	96.7 (15.9) (*n* = 6)	89.4 (9.0) (*n* = 5)	0.47	82.3 (3.2) (*n* = 3)	97.5 (13.1) (*n* = 8)	0.07
%FEV_1_ (%)	83.2 (22.8)	71.7 (19.9)	0.18	64.0 (12.9)	85.6 (22.2)	0.024^†^
—MEPO (−) (*n* = 13)	86.2 (20.6) (*n* = 8)	69.5 (28.6) (*n* = 5)	0.38	60.5 (14.3) (*n* = 5)	91.8 (25.2) (*n* = 8)	0.028^†^
—MEPO (+) (*n* = 11)	79.1 (13.1) (*n* = 6)	74.0 (21.3) (*n* = 5)	0.47	69.7 (9.5) (*n* = 3)	79.4 (18.4) (*n* = 8)	0.41
comorbidities						
—with AERD, n (%)	2 (14)	5 (50)	0.085	3 (38)	4 (25)	0.65
—with ECRS, n (%)	12 (86)	8 (80)	> 0.99	7 (88)	13 (81)	> 0.99

Data at baseline are presented as mean (standard deviation), unless otherwise stated

*P* value was analyzed using Fisher’s exact test or Mann-Whitney U test

*Abbreviations*: *GETE* global evaluation of treatment effectiveness, *ΔFEV*_*1*_ change from baseline to the last follow-up in the forced expiratory volume in 1 s, *MEPO* previous mepolizumab treatment, *BMI* body mass index, *bEOS* peripheral blood eosinophil count at baseline, *bBASO* peripheral basophil count at baseline, *FeNO* fractional exhaled nitric oxide, *FVC* forced vital capacity, *FEV1* forced expiratory volume in 1 s, *AERD* aspirin-exacerbated respiratory disease, *ECRS* eosinophilic chronic rhinosinusitis, *ppb* parts-per-billion

^†^*P* values with sufficient power that were re-evaluated by a post-hoc power analysis

^a^data missing (*n* = 1)

### Analysis of clinical characteristics using multivariate logistic regression

We performed subgroup analyses using univariate logistic regression of the GETE score as follows: 1) patient characteristics and parameters, 2) with or without previous mepolizumab treatment, 3) with or without AERD as a comorbidity, and 4) with or without ECRS as a comorbidity (Table 4). Then, we selected the peripheral blood eosinophil count (≥ 300 /μl), ECRS or CRSwNP, AERD, FeNO (≥ 50 parts-per-billion) and BMI (≥ 25 kg/m^2^) for multivariate logistic regression analysis of the GETE score. We found that the number of patients with AERD was significantly lower in the GETE responder group than in the non-responder group [odds ratio (OR) 0.035, 95% confidence interval (CI) (0.002–0.72), *p* = 0.03] (Table 4).

**Table 4:** Clinical characteristics in the GETE score using a univariate and multivariate logistic regression

	GETE ≥ good (*n* = 14)	GETE ≤ moderate (*n* = 10)	odds ratio (95%CI) (univariate)	*p* value	odds ratio (95%CI) (multivariate)	*p* value
sex (male), n (%)	4 (29)	5 (50)	0.40 (0.07–2.2)	0.29	–	–
age (≥ 65 year-old), n (%)	5 (36)	4 (40)	0.83 (0.16–4.4)	0.83	–	–
BMI (≥ 25) (kg/m^2^), n (%)	3 (21)	5 (50)	0.27 (0.05–1.6)	0.15	0.1 (0.004–2.8)	0.18
bEOS (≥ 300) (/μl), n (%)	7 (50)	3 (30)	2.3 (0.42–12.9)	0.33	11.2 (0.57–219)	0.11
bBASO (≥ 40) (/μl), n (%)	4 (29)	3 (30)	0.93 (0.16–5.5)	0.94	–	–
FeNO (≥ 50) (ppb), n (%)	9 (64)	3 (30)	3.6 (0.62–21)	0.15	3.7 (0.24–57)	0.35
MEPO (+), n (%)	6 (43)	5 (50)	0.75 (0.15–3.8)	0.73	–	–
comorbidities						
—with AERD, n (%)	2 (14)	5 (50)	0.17 (0.02–1.2)	0.07	0.035 (0.002–0.72)	0.03
—with ECRS, n (%)	12 (86)	8 (80)	1.5 (0.17–13)	0.71	0.51 (0.006–41)	0.76

*Abbreviations*: *GETE* Global Evaluation of Treatment Effectiveness, *BMI* body mass index, *bEOS* peripheral blood eosinophil count at baseline, *bBASO* peripheral basophil count at baseline, *FeNO* fractional exhaled nitric oxide, *ppb* parts-per-billion, *MEPO* previous mepolizumab treatment, *AERD* aspirin-exacerbated respiratory disease, *ECRS* eosinophilic chronic rhinosinusitis

### Others

Four patients discontinued benralizumab treatment for the short term for the following reasons: adverse effects [headache (*n* = 1) and injection site pain (*n* = 1)], poor effectiveness for asthma (*n* = 1) and other (*n* = 1) (data not shown).

We evaluated sinusitis symptoms in 18 of the 20 patients with comorbid SEA and ECRS. Based on patient symptoms in the medical records, nine patients (50%) reported the efficacy of benralizumab treatment for sinusitis (data not shown).

## Discussion

This retrospective, real-life benralizumab study showed that the response rate based on the GETE score was lower than the rate reported in real-life mepolizumab studies [11, 16]. The number of annual exacerbations and maintenance CS doses tended to improve in patients without previous mepolizumab treatment. These parameters did not significantly decrease unlike those reported in major clinical trials and in Japanese subgroups [6, 8, 9, 12]. These findings may be due to several reasons. First, real-life studies [11, 21, 22] generally enroll a relatively large number of patients with prior use of biologics compared to major RCTs [6, 8] (13–33% vs 3–8% of patients). Hence, real-life studies may likely include a higher proportion of patients with treatment-refractory asthma. Accordingly, we speculate that the lower response rate based on the GETE score can be attributed to a higher proportion of patients who needed to switch biologics in the present study. Intriguingly, however, 75% of the patients had maintained or improved asthma symptoms after changing to benralizumab in the present study, indicating the potential usefulness of benralizumab in the setting of switching biologics (Additional file 1).

Second, the proportion of all enrolled patients with AERD was approximately 30%. In general, the prevalence of AERD is 7% in adult asthma populations and 15% in severe asthma populations [23]. In general, AERD is associated with weak pulmonary function and is also more likely to be associated with severe asthma [23]. Consistent with a previous retrospective study showing poor improvement of the FEV_1_ in patients with AERD after mepolizumab treatment [24], we showed no significant difference in the %FEV_1_ before and after treatment in the AERD group. Third, we hypothesized that seasonal asthmatic exacerbations influenced the efficacy of benralizumab treatment. In the present study, only 50% of all patients received benralizumab treatment for 12 months or longer. Accordingly, seasonal variability may have led to these results [25]. Fourth, the efficacy of benralizumab might not differ from that of mepolizumab despite the complete disappearance of peripheral blood eosinophils. Several indirect meta-analyses have investigated these biologics, but the results are controversial [26–28].

The ACT score and %FEV_1_ in the AERD group were lower than those in the AERD (−) group, and a significant difference in the GETE score was identified between the two groups. Few reports on the effectiveness of biologics in patients with AERD are available. A previous study reported that omalizumab displayed rapid clinical effectiveness and inhibited mast cell activation and leukotriene overproduction in AERD [29, 30]. Although mepolizumab treatment resulted in significant improvement of asthma and nasal symptoms in AERD in a retrospective study, the %FEV_1_ did not improve [24]. To our knowledge, no similar results regarding benralizumab are available. The pooled analysis of RCTs demonstrated that nasal polyps were predictors of the response to benralizumab in SEA [7, 31]. However, clinical differences have also been reported to exist between patients with asthma and ECRS and those with AERD, such as differences in pulmonary function, the prevalence of OCS treatment and cytokine levels in nasal polyps [32, 33]. Furthermore, a previous report demonstrated that a functional polymorphism in IL5RA may contribute to eosinophil and mast cell activation along with specific IgE responses to staphylococcal enterotoxin A in AERD patients [34]. These findings indicate that anti-IgE antibody may be more effective than anti-IL-5/IL-5RA antibody, or that the drug response to anti-IL-5/IL-5RA antibody may be different in some patients with AERD. We need further data from patients with AERD receiving anti-IL-5/IL-5RA antibody treatment.

We showed that switching from mepolizumab to benralizumab tended to slightly improve the mean values of some parameters without significant differences, but three patients demonstrated a change in FEV_1_ ≥ 200 ml. On the other hand, the number of exacerbations increased without a significant difference, and one patient with AERD was switched backed to mepolizumab (Additional file 1). Further investigation is needed to determine the proper use of these two biologics.

Several limitations to the present study exist. First, this was a small, single-center, retrospective study. However, since the number of patients who received benralizumab (Q8W) in the Japanese subgroup of the CALIMA trial was 15, analyzing small-group studies such as these is also important. Thus, to further confirm the primary statistical measures, we reevaluated the results via post-hoc power analysis. Second, the duration of benralizumab treatment was short (mean 11.5 months, range 4–17 months). Although the SIROCCO trial [6], a representative clinical trial of benralizumab, showed the efficacy of benralizumab within 4 months and the GINA guideline [3] recommends assessing the efficacy of biologics at approximately 4 months, we speculate that further long-term observations are required not only to precisely evaluate the efficacy but also to elucidate response predictors in a real-life study including patients with diverse backgrounds. Third, in the present study, 46% of the patients switched therapies. However, we showed interesting results that were not found in previous RCTs following the switch from mepolizumab. As the future studies, prospective multicentered clinical trials with more cases are necessary to verify the present results.

## Conclusion

Benralizumab treatment for patients with SEA showed a clinical efficacy of approximately 60% based on the GETE score and may significantly improve the FEV_1_ in some patients with previous mepolizumab treatment.

## Supplementary information

**Additional file 1.** Characteristics of 12 patients who received other biologics before benralizumab treatment

**Additional file 2.** Patients characteristics at baseline in four subgroup [male, female, AERD (−) and (+)]

**Additional file 3 Fig.** Changes in clinical parameters in the AERD (−) and (+) groups. All results are expressed as individual data, and the boxes represent the median and interquartile ranges. The upper and lower whiskers represent the 90th and 10th percentiles, respectively. These data were analyzed with the Mann-Whitney *U* test or the Wilcoxon signed rank test. A: No significant differences in ACT scores were found before and after treatment in each group (Wilcoxon signed rank test). However, a significant difference was found in ACT scores at baseline between the two groups (*p* = 0.012, Mann-Whitney *U* test). ^†^*P* values with sufficient power that were re-evaluated by a post-hoc power analysis. B: Significant differences in the %FEV_1_ were found before and after treatment in the AERD (−) group (*p* = 0.044, Wilcoxon signed rank test). Furthermore, significant differences in the %FEV_1_ at baseline and after treatment were identified between the two groups (*p* = 0.028 and *p* = 0.031, respectively, Mann-Whitney *U* test). P values without sufficient power that were re-evaluated by a post-hoc power analysis. Abbreviations: ACT; Asthma Control Test, AERD; aspirin-exacerbated respiratory disease, %FEV_1_; % forced expiratory volume in 1 s

## Data Availability

The datasets used and/or analyzed during the current study are available from the corresponding author upon reasonable request.

## Abbreviations

ACT: Asthma Control Test
AERD: Aspirin-exacerbated respiratory disease
BMI: Body mass index
CRSwNP: Chronic rhinosinusitis with nasal polyps
CS: Corticosteroid
ECRS: Eosinophilic chronic rhinosinusitis
GETE: Global Evaluation of Treatment Effectiveness
ICS: Inhaled corticosteroid
IL: Interleukin
LABA: Long-acting β-2 agonist
LAMA: Long-acting muscarinic antagonist
LTRA: Leukotriene receptor antagonist
RCT: Randomized control trial
SEA: Severe eosinophilic asthma
