# Supplementary figures and images for: Efficacy of benralizumab for patients with severe eosinophilic asthma: a retrospective, real-life study
Source: BMC Pulm Med. 2020 Aug 3;20:207. doi: 10.1186/s12890-020-01248-x (PMC7398222; doi:10.1186/s12890-020-01248-x)

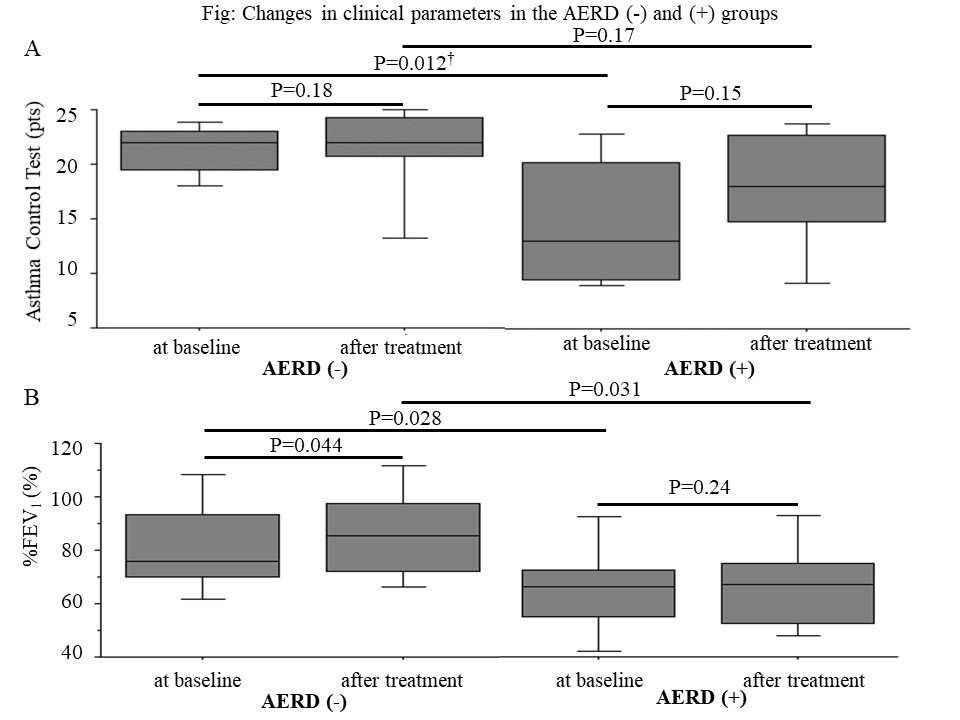

Supplement: Supplementary file 3 — Additional file 3 Fig. Changes in clinical parameters in the AERD (−) and (+) groups. All results are expressed as individual data, and the boxes represent the median and interquartile ranges. The upper and lower whiskers represent the 90th and 10th percentiles, respectively. These data were analyzed with the Mann-Whitney U test or the Wilcoxon signed rank test. A: No significant differences in ACT scores were found before and after treatment in each group (Wilcoxon signed rank test). However, a significant difference was found in ACT scores at baseline between the two groups (p = 0.012, Mann-Whitney U test). †P values with sufficient power that were re-evaluated by a post-hoc power analysis. B: Significant differences in the %FEV1 were found before and after treatment in the AERD (−) group (p = 0.044, Wilcoxon signed rank test). Furthermore, significant differences in the %FEV1 at baseline and after treatment were identified between the two groups (p = 0.028 and p = 0.031, respectively, Mann-Whitney U test). P values without sufficient power that were re-evaluated by a post-hoc power analysis. Abbreviations: ACT; Asthma Control Test, AERD; aspirin-exacerbated respiratory disease, %FEV1; % forced expiratory volume in 1 s [file 12890_2020_1248_MOESM3_ESM.jpg]
